# Dietary Glucose Oxidase Supplementation During Gestation Improves Health Status by Affecting Antioxidant Capacity, Immune Function, and Gut Microbiota of Farrowing Sows

**DOI:** 10.3390/microorganisms14051005

**Published:** 2026-04-29

**Authors:** Shuning Zhang, Xiaomin Wang, Guifeng Zhang, Lei Kong, Yuemeng Fu, Guohui Zhou, Qingsong Fan, Zhenhui Liu, Shuzhen Jiang, Yang Li

**Affiliations:** 1 Key Laboratory of Efficient Utilization of Non-Grain Feed Resources (Co-Construction by Ministry and Province), Ministry of Agriculture and Rural Affairs, Shandong Provincial Key Laboratory of Animal Nutrition and Efficient Feeding, College of Animal Science and Technology, Shandong Agricultural University, Panhe Street 7, Tai’an 271017, China; zsn815@126.com (S.Z.); fuyuemeng@yeah.net (Y.F.); zhou_guohui@yeah.net (G.Z.); fan_qingsong@yeah.net (Q.F.); liu_zhenhui@yeah.net (Z.L.); szjiang@sdau.edu.cn (S.J.); 2 Shandong Hemeihua Biotechnology Co., Ltd., Jinan 250104, China; xiaomin616@yeah.net; 3 Shandong Lonct Enzymes Co., Ltd., Linyi 276400, China; zgf1122@163.com; 4 Shandong Provincial Animal Husbandry General Station (Shandong Provincial Quality Testing Station for Breeding Livestock and Poultry), Jinan 250109, China; kongleiisme@163.com

**Keywords:** antioxidant capacity, gut microbiota, glucose oxidase, inflammation, placenta, sows

## Abstract

Glucose oxidase (GOD) is a natural enzyme with antioxidant and antimicrobial properties but its effects on sows remain insufficient. This study investigated the effects of dietary GOD supplementation during gestation on inflammatory response, antioxidant capacity, immune function, and gut microbiota of farrowing sows. Twenty-four primiparous sows were randomly assigned to two groups and fed a basal diet or a basal diet supplemented with GOD (300 mg/kg diet) from gestation day 30 to farrowing. GOD supplementation significantly increased triglyceride, superoxide dismutase, and immunoglobulin M levels (*p* < 0.05), and significantly decreased alanine aminotransferase and interleukin-6 levels in serum (*p* < 0.05); significantly reduced placental interleukin-1β, malondialdehyde and tumor necrosis factor-α concentrations and *NF-κB* gene expression (*p* < 0.05), and elevated glutathione peroxidase activity and relative mRNA expressions of *Nrf2*, *HO-1*, *GPX1* and *SOD2* (*p* < 0.05). Moreover, GOD supplementation altered the fecal microbial community structure (*p* < 0.05), significantly reducing *Clostridium*, *dgaA-11_gut_group*, *Bacteroides*, and *Prevotellaceae_NK3B31_group* abundance (*p* < 0.05), while enriching *Lachnospira*, *unclassified_f_Erysipelotrichiaceae*, and *Anaerostipes* (*p* < 0.05). Collectively, 300 mg/kg glucose oxidase supplementation during mid-to-late gestation improved the health status of farrowing sows by improving nutrient utilization, immune function and antioxidant capacity, and altering fecal microbial structure and relative abundances.

## 1. Introduction

To enhance production efficiency, modern pig farming has increased litter sizes through genetic selection, which imposed sustained metabolic and physiological stress on sows [1]. From day 30 of gestation to farrowing, sows experience endocrine fluctuations accompanied by elevated metabolic demands due to rapid fetal growth, placental maturation, and preparation for lactation [2]. In primiparous sows, the requirements for maternal growth and reproductive nutrition allocation further exacerbate metabolic burden. Excessive metabolic load can disrupt the balance between reactive oxygen species (ROS) generation and the antioxidant defense system, leading to oxidative stress [3,4]. Persistent oxidative stress activates immune suppression and exacerbates inflammatory responses, resulting in elevated pro-inflammatory cytokines such as tumor necrosis factor-α (TNF-α) and interleukin-6 (IL-6) [3,5]. Oxidative stress and inflammation compromise not only the maternal health but also placental function through systemic circulation, adversely affecting fetal development and the establishment of fetal physiological and immune homeostasis [6,7]. In addition, peripartum stress has also been reported to disturb intestinal microbiota in sows [8]. This facilitates the translocation of harmful substances, including endotoxins, into systemic circulation, further amplifying systemic inflammation and oxidative stress [8]. Hormonal fluctuations peak at farrowing, and prolonged labor, particularly in large litters, can cause irreversible reproductive system damage and increased susceptibility to postpartum infections, which contribute to culling rates [1,9]. Therefore, mitigating peripartum period metabolic and farrowing stress is critical for maintaining sows’ health.

In the past, antibiotics were commonly added to feed to reduce the farrowing stress of sows. However, given that antibiotic resistance and drug residues in animal products pose a significant threat to public health, the development of antibiotic alternatives has become an urgent priority in animal husbandry [10]. Various feed additives, including plant extracts, organic acids, enzymes and probiotics, have been used to alleviate peripartum stress in sows [11]. Glucose oxidase (GOD), a microbial and insect-derived enzyme, exerts its redox-regulating and antimicrobial effects through multiple mechanisms. It catalyzes the oxidation of β-D-glucose into gluconic acid and a nontoxic concentration of hydrogen peroxide (H_2_O_2_) while consuming oxygen [12]. The produced gluconic acid lowers intestinal pH, creating an environment unfavorable for pathogenic bacteria, while H_2_O_2_ acts as a signaling molecule to activate the host antioxidant defense system, thereby contributing to both antimicrobial and antioxidant effects [12]. Moreover, GOD can be produced cost-effectively on a large scale via microbial fermentation, providing a more economically viable alternative to other specialized feed additives [13]. Due to these properties, GOD has been recognized as a functional additive for alleviating oxidative stress, maintaining animal health, and promoting growth [12]. Accumulating evidence from previous studies has confirmed the beneficial effects of GOD in different animal species. For instance, dietary supplementation with 100, 300 and 500 U/kg GOD was shown to mitigate weaning stress, intestinal damage, and gut microbiota dysbiosis, and enhance growth performance in weaned piglets for 21 days [14]. Dietary supplementation with 200 g/t GOD for 21 days alleviated intestinal injury in enterotoxigenic Escherichia coli-challenged weaned piglets [15]. Studies in broilers have shown that GOD supplementation at 100, 200 and 300 mg/kg for 35 days increased the content of secreted immunoglobulin A (IgA), thereby enhancing gut barrier function [16]. Meanwhile, blood antioxidant indices, including GSH-Px and glutathione, were also linearly elevated with the increased supplementation levels of GOD in broilers [16]. In multiparous sows, supplementing 60 U/kg GOD to the diet during the entire gestation and lactation periods also showed positive effects on improving reproductive performance, enhancing antioxidant capacity and inhibiting harmful bacteria Escherichia coli growth during lactation [17]. Sureshkumar et al. [18] supplemented 200, 300, and 400 U/kg GOD in the diets of late gestation and lactating sows and observed linear improvement in nutrient digestibility and blood profile in lactating sows. Collectively, these findings suggest that GOD can contribute to improving animal health and reducing the reliance on antibiotics in livestock production. However, studies on its application in sows remain insufficient.

Based on the above, we hypothesized that dietary GOD supplementation from mid-to-late gestation would improve the blood profile, alleviate inflammation and oxidative stress, and modulate the gut microbiota. Therefore, this study aimed to evaluate the impacts of dietary GOD supplementation from day 30 of gestation to farrowing on the nutrient metabolism, immune response, antioxidant status and gut microbiota of sows, providing theoretical support for GOD to alleviate delivery stress in farrowing sows.

## 2. Materials and Methods

### 2.1. Animal Management and Experiment Design

A total of 24 Large White × Landrace crossbred primiparous sows at gestation day 30 (265 ± 1 days of age, 161.19 ± 3.37 kg body weight, and 15.69 ± 0.64 mm backfat thickness), which had been artificially inseminated with semen from Yorkshire boars, were employed in this study. All sows were fed the same basal diet from gestating day 0 to 29, and the detection of pregnancy was 26 days after being inseminated. On day 30 of gestation, the 24 pregnant sows were assigned randomly to two groups (12 replicates per group, one sow per replicate): the control group (CON) fed the basal diet, and the GOD group fed the basal diet supplemented with 300 mg GOD product per kg of diet. The GOD used in the study was provided by Shandong Lonct Enzymes Co., Ltd. (Linyi, China), and the activity of GOD in the product is 2000 U/g. The GOD dosage was selected based on the dose-dependent linear effects reported in the previous study [18]. The basal diet (Table 1) was formulated according to the National Research Council (NRC, 2012) [19] to meet or exceed the nutrient requirements of gestating sows. Diets were provided twice daily at 8:00 and 16:00, with ad libitum access to water. Sows received 2.30 kg of diet from day 30 to 90, and 2.60 kg from day 91 to farrowing. On day 110 of gestation, the sows were transferred from group pens to farrowing crates.

### 2.2. Sample Collection

On day 113 of gestation, fresh fecal samples were collected from 12 sows via rectum into sterile tubes, immediately snap-frozen in liquid nitrogen, and stored at −80 °C for subsequent 16S rRNA gene sequencing analysis [20]. On the day of farrowing, blood samples were collected from 6 randomly selected sows per group via the ear vein using sterile vacuum blood collection tubes [12]. Subsequently, all blood samples were centrifuged at 4000 rpm for 10 min at 4 °C to obtain serum and stored at −20 °C until analysis. Placental samples were collected within 30 min after complete placental expulsion, rinsed with 0.9% saline to remove residual blood and mucus, and then stored at −80 °C until further analysis.

### 2.3. Serum Biochemical Analysis

Serum biochemical parameters associated with nutrient metabolism and liver function, including glucose (GLU), cholesterol (CHOL), triglyceride (TG), total protein (TP), blood urea nitrogen (BUN), albumin (ALB), aspartate aminotransferase (AST), alanine aminotransferase (ALT), alkaline phosphatase (ALP), and lactate dehydrogenase (LDH), were performed using Cobas Mira Plus Automatic Biochemical Analyzer (Roche, Montclair, NJ, USA).

### 2.4. Serum Immunoglobulin Determination

The concentrations of IgA, IgM, and IgG in serum were measured using commercial ELISA kits (Jiangsu Meimian Industrial Co., Ltd., Yancheng, China). All procedures were conducted according to the protocol previously described by Chen et al. [10].

### 2.5. Antioxidant Indices and Inflammatory Cytokines Determination

Placental samples were homogenized in 0.9% saline at a ratio of 1:9 (*w*/*v*) and centrifuged to obtain supernatant. Serum and placental supernatant samples were analyzed for antioxidant indices and inflammatory cytokine concentrations. The antioxidant indices, including superoxide dismutase (SOD), GSH-Px, CAT, and MDA, were determined using commercial kits obtained from Nanjing Jiancheng Bioengineering Institute (Nanjing, China) according to the manufacturer’s instructions.

The inflammatory cytokines, including interleukin-1β (IL-1β), IL-6, TNF-α, and interleukin-10 (IL-10), were determined using ELISA kits specific for swine (Jiangsu Meimian Industrial Co., Ltd., Yancheng, China), following the protocol previously described by Chen et al. [10]. The results of placental parameters were normalized to total protein content.

### 2.6. Real-Time PCR

Total RNA of the placenta was extracted using AIPzol Reagent (I-presci Scientific, Beijing, China). RNA was reverse-transcribed into cDNA using an Evo M-MLV Reverse Transcription Premix Kit (Accurate Biology, Changsha, China) according to the manufacturer’s instructions. All primers were synthesized commercially by Sangon Biotech Limited and primer sequences are listed in Table 2. Quantitative real-time PCR was performed to analyze the expression levels of nuclear factor erythroid-derived 2-like 2 (*Nrf2*), glutathione peroxidase 1 (*GPX1*), catalase (*CAT*), superoxide dismutase 1 (*SOD1*), superoxide dismutase 2 (*SOD2*), nuclear factor kappa-B (*NF-κB*), and heme oxygenase-1 (*HO-1*) with the SYBR Green Pro Taq HS Premix qPCR Kit (Accurate Biology, Changsha, China) according to the manufacturer’s instructions. The β-actin gene was used as the internal reference gene. All reactions were performed in triplicate with three biological replicates, and the relative expression levels of the target genes were calculated using the 2^−ΔΔCt^ method.

### 2.7. 16S rRNA Analysis of Fecal Microbiota

Total genomic DNA was extracted from the fecal samples using a commercial fecal DNA extraction kit (Omega Bio-tek, Norcross, GA, USA) following the manufacturer’s instructions. Then the quality and concentration of DNA were measured using 1% agarose gel electrophoresis and a NanoDrop 2000 UV–vis spectrophotometer (Thermo Scientific, Waltham, MA, USA). The V3-V4 region of the 16S rRNA gene was amplified by polymerase chain reaction (PCR) using primers 338F (ACTCCTACGGGAGGCAGCAG) and 806R (GGACTACHVGGGTWTCTAAT). After purifying and quantifying the PCR product, high-throughput sequencing of bacterial 16S rRNA was conducted on the Illumina Nextseq2000 platform. Raw sequence data were processed by fastp (0.23.4), and low-quality sequences were eliminated [21]. Sequencing errors and chimeras were eliminated using USEARCH (v2.17.8) [22]. The remaining sequences were clustered into operational taxonomic units (OTUs) at 97% similarity against the Silva v138 database [22]. Data were analyzed through the Majorbio Cloud (Shanghai, China). Alpha indices, including the abundance-based coverage estimator (ACE), Chao1, Shannon, and Simpson indexes, were analyzed using Mothur (v1.48.3) [23]. Principal coordinate analysis (PCoA) was employed to assess beta diversity based on unweighted_unifrac distance metrics in Mothur (v1.48.3) [24]. Linear discriminant analysis effect size (LEfSe) was employed to identify differences in bacterial taxa between groups. The visualization was performed using R software (v3.3.1).

### 2.8. Statistical Analysis

All experimental data were organized using Microsoft Excel 2019 (Microsoft Corporation, Redmond, WA, USA). An independent sample t-test was employed to compare physiological indices between the two groups, using IBM SPSS Statistics 22.0. Microbiota composition differences were assessed using the Wilcoxon test.

Prior to the *t*-test, the normality of each indicator dataset was verified via the Shapiro–Wilk test. If the data were normally distributed, Levene’s test was used to assess the homogeneity of variances between the two groups. The standard independent samples *t*-test was applied when Levene’s test yielded a *p*-value > 0.05 (satisfying the variance homogeneity assumption); whereas the Welch-corrected independent samples *t*-test was adopted when *p* ≤ 0.05 (indicating variance heterogeneity). A two-tailed significance level of α = 0.05 was set for all statistical analyses. A *p*-value < 0.05 was considered statistically significant.

## 3. Results

### 3.1. Effects of GOD Supplementation During Gestation on Serum Biochemistry of Farrowing Sows

As shown in Table 3, the concentration of ALT was significantly decreased in the GOD group compared with the CON group (*p* < 0.05). The concentration of TG was significantly increased in the GOD group compared with the CON group (*p* < 0.05). Additionally, the concentration of BUN tended to be higher in the GOD group (*p* < 0.10). No significant differences were observed in the other indices (*p* > 0.05).

### 3.2. Effects of GOD Supplementation During Gestation on Serum Immunoglobulins of Farrowing Sows

The effects of dietary GOD supplementation on serum immune indicators in sows are shown in Table 4. The concentration of IgM was significantly increased in the GOD group compared with the CON group (*p* < 0.05). However, no significant differences were observed in serum IgA or IgG levels between the two groups (*p* > 0.05).

### 3.3. Effects of GOD Supplementation During Gestation on Serum and Placental Inflammatory Cytokine Levels of Farrowing Sows

As presented in Table 5, in serum, the concentration of IL-6 was significantly reduced in the GOD group relative to the CON group (*p* < 0.05), while IL-10 concentrations were significantly elevated (*p* < 0.05). There were no significant differences in serum IL-1β and TNF-α concentrations between the two groups (*p* > 0.05). In placental tissue, IL-1β and TNF-α concentrations were significantly decreased in the GOD group compared with the CON group (*p* < 0.05). There were no significant differences in placental IL-6 and IL-10 concentrations between the two treatments (*p* > 0.05).

### 3.4. Effects of GOD Supplementation During Gestation on Serum and Placental Antioxidant Capacities of Farrowing Sows

Results of serum and placental antioxidant capacities related parameters in sows are shown in Table 6. In serum, the GOD group had significantly higher SOD activity than the CON group (*p* < 0.05). The activities of GSH-Px and CAT, and MDA level did not significantly differ between the GOD and CON groups (*p* > 0.05). In the placenta, the GSH-Px activity in the GOD group significantly increased compared with the CON group (*p* < 0.05), while the MDA concentration was significantly decreased (*p* < 0.05). No significant differences were observed in the CAT and SOD activities between the two groups (*p* > 0.05).

### 3.5. Effects of GOD Supplementation During Gestation on Relative mRNA Expression in the Placenta of Farrowing Sows

**Figure 1:**
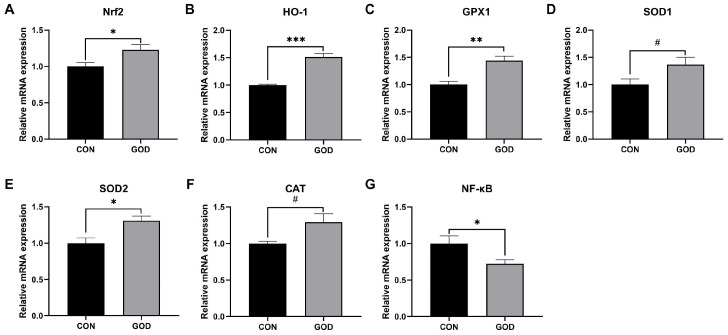
Effects of glucose oxidase (GOD) supplementation on relative mRNA expression in the placenta of farrowing sows. CON, sows fed a basal diet; GOD, sows fed a basal diet supplemented with 300 mg/kg GOD. (**A**) *Nrf2*, nuclear factor erythroid 2 related factor 2; (**B**) *HO-1*, heme oxygenase-1; (**C**) *GPX1*, glutathione peroxidase-1; (**D**) *SOD1*, superoxide dismutase 1; (**E**) *SOD2*, superoxide dismutase 2; (**F**) *CAT*, catalase; (**G**) *NF-κB*, nuclear factor-kappa B. Data are expressed as mean ± standard error of the mean (SEM) with *n* = 6 per group. Statistical significance is indicated as follows: ^#^ 0.05 ≤ *p* < 0.10, * *p* < 0.05, ** *p* < 0.01, *** *p* < 0.001.

The relative mRNA expressions in the placenta of sows are displayed in Figure 1. Dietary supplementation with 300 mg/kg GOD significantly increased the relative mRNA expressions of *Nrf2*, *HO-1*, *GPX1*, and *SOD2* (*p* < 0.05) and significantly decreased the relative mRNA expressions of *NF-κB* (*p* < 0.05) in the placenta. Moreover, 300 mg/kg GOD supplementation tended to increase the relative mRNA expressions of *SOD1* and *CAT* in the placenta (*p* < 0.10).

### 3.6. Effects of GOD Supplementation During Gestation on Fecal Microbiota of Farrowing Sows

#### 3.6.1. Diversity and Structure of Fecal Microbial Communities

**Figure 2:**
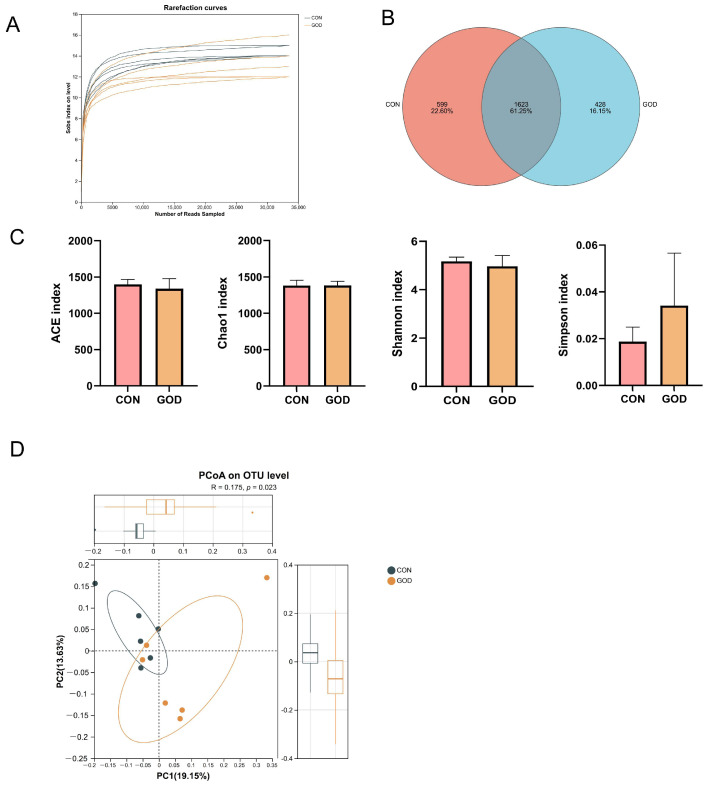
Effects of glucose oxidase (GOD) supplementation on fecal microbiota richness and diversity. CON, sows fed a basal diet; GOD, sows fed a basal diet supplemented with 300 mg/kg GOD. (**A**) Rarefaction curve; (**B**) Venn diagram; (**C**) alpha diversity indexes, including ACE, Chao 1, Shannon, Simpson index; (**D**) principal co-ordinates analysis (PCoA) plot. *n* = 6.

As shown in Figure 2A, the rarefaction curves approached a plateau, indicating that the sequencing depth was sufficient to cover the species diversity of all 12 samples. The Venn diagram showed that the CON group contained 2222 OTUs, while 2051 OTUs were detected in the GOD group. Of these, 1623 OTUs were shared between the two groups, with 599 OTUs unique to the CON group and 428 OTUs exclusive to the GOD group (Figure 2B). There were no statistically significant differences in the ACE index, Chao1 index, Shannon index, and Simpson index (Figure 2C) between the two groups (*p* > 0.05). In addition, the PCoA plot based on unweighted_unifrac distance showed that the bacterial structure in feces between the two groups was statistically significant (*p* = 0.023, R = 0.175; Figure 2D).

#### 3.6.2. Fecal Microbial Composition and Differences

**Figure 3:**
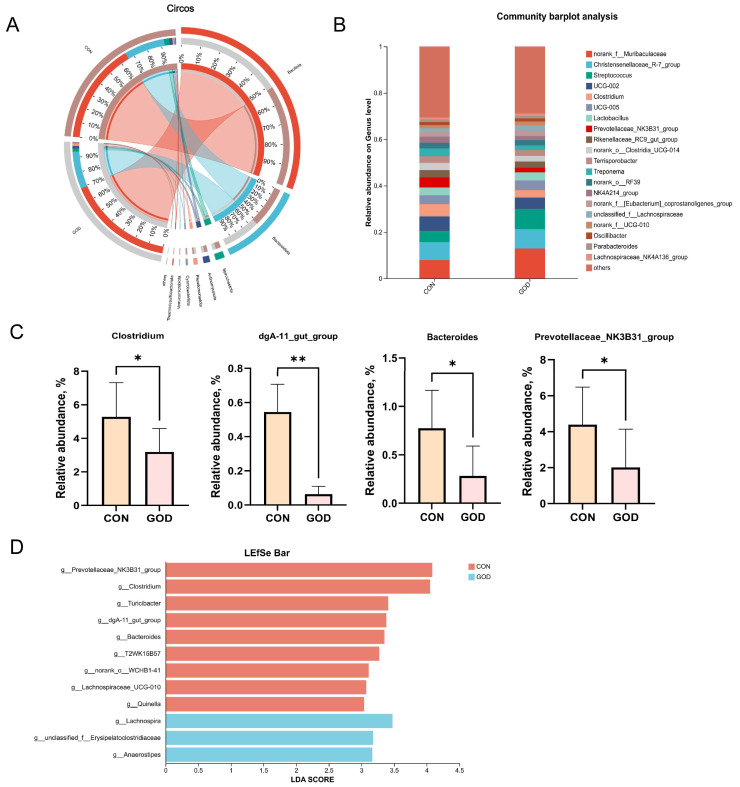
Effects of GOD supplementation on fecal microbiota composition and genus-level differences in farrowing sows. CON, sows fed a basal diet; GOD, sows fed a basal diet supplemented with 300 mg/kg GOD. (**A**) Circos plot of the fecal microbiota composition at the phylum level (top 10); (**B**) histograms of the fecal microbiota composition at the genus level (top 25); (**C**) bar plot of the relative abundance of genera with significant differences; (**D**) linear discriminant analysis effect size (LEfSe) bar plot of differential microbiota between groups with threshold 3. *n* = 6. * indicates *p* <0.05, and ** indicates *p* < 0.01.

The Circos plot (Figure 3A) shows the relative abundance of the top 8 phyla (Bacillota, Bacteroidota, Spirochaetota, Actinomycetota, Pseudomonadota, Cyanobacteriota, Verrucomicrobiota and Thermodesulfobacteriota). The dominant phyla in the CON and GOD groups were Bacillota and Bacteroidota. At the genus level (top 25), *norank_f__Muribaculaceae*, *Christensenellaceae_R-7_group*, and *Streptococcus* were the dominant genera (Figure 3B). The relative abundances of *Clostridium*, *dgaA-11_gut_group*, *Bacteroides*, and *Prevotellaceae_NK3B31_group* were significantly reduced in the GOD group compared to the CON group (Figure 3C, *p* < 0.05). This reduction was also identified by LEfSe analysis (Figure 3D, LDA score ≥ 3). Moreover, LEfSe analysis revealed that *Turicibacter*, *T2WK15B57*, *norank_o_WCHB1-41*, *Lachnospiraceae_UCG-010*, and *Quinella* enriched in the CON group, while *Lachnospira*, *unclassified_f_Erysipelotrichiaceae*, and *Anaerostipes* enriched in the GOD group.

## 4. Discussion

Serum biochemical parameters serve as important biomarkers for evaluating nutritional metabolic status and organ function in animals [25]. In this study, GOD supplementation elevated the serum concentrations of TG and BUN of farrowing sows. TG is one of the only two major energy substrates that the uterus actively extracts from the blood during farrowing [26]. Higher TG levels are associated with shorter farrowing duration, thereby decreasing the risk of postpartum infection [27]. BUN, produced by protein catabolism, serves as an indicator of amino acid balance [28]. Increased metabolism of dietary amino acids and proteins elevated serum BUN [29,30,31]. A previous study demonstrated that dietary GOD supplementation enhanced apparent nitrogen digestibility in piglets [32], which could be the reason for the increased serum BUN [30]. Therefore, the higher TG and BUN might indicate the effective energy and protein preparation for farrowing in primiparous sows. Furthermore, we also found decreased serum ALT activity in sows supplemented with GOD. ALT is a reliable indicator of hepatocellular membrane integrity, and its elevated levels in serum are associated with liver injury [33]. Farrowing has been reported to increase stress in sows, accompanied by elevated hepatic and renal enzymes, including AST, ALP and LDH, in saliva [34], indicating hepatic and renal injury induced by farrowing stress. Wang et al. [15] found that GOD decreased ALT in Escherichia coli-challenged piglets, demonstrating the protective effects of GOD on the liver. Sun et al. [17] also showed that dietary supplementation with 75 U/kg GOD attenuated liver injury by reducing hepatocyte apoptosis in multiparous sows at weaning. Therefore, the reduced serum ALT level indicated the potential to alleviate hepatic stress in sows at farrowing. Collectively, supplementing gestating sows with 300 mg/kg GOD positively influenced nutritional metabolism and hepatic function for gestating sows and supporting farrowing.

Immunoglobulins are essential components of the humoral immune system, and their concentrations reflect the immune status of animals [35]. The present study indicated that GOD supplementation significantly increased serum IgM levels. IgM, the first antibody produced during the primary immune response, plays a crucial role in pathogen combat, immune complex clearance, and complement activation [36]. Previous studies also showed that GOD could improve immune status by increasing serum immunoglobulin levels in weaned piglets and ducks [10,37]. Sows are subject to intense stress and infection risk during the perinatal and farrowing periods. Therefore, the elevation of IgM might imply a stronger immune function to mitigate stress-induced inflammatory responses and reduce susceptibility to infections [38]. The results suggested evidence for the immunomodulatory effect of GOD in improving the humoral immune competence of farrowing sows. Enhanced immune competence is closely associated with attenuated inflammatory responses in gestating sows, as supported by numerous dietary interventions that effectively strengthen immune status while mitigating systemic and intestinal inflammation. Inflammatory cytokines regulate both systemic and local immune responses, and the balance between pro- and anti-inflammatory cytokines is essential for animal health [39]. Our study showed that dietary GOD supplementation not only decreased IL-6 levels and elevated IL-10 concentrations in serum but also decreased the levels of IL-1β and TNF-α in the placenta. IL-6, IL-1β, and TNF-α are pro-inflammatory cytokines involved in infection- and injury- induced inflammatory responses, whereas IL-10 functions as a key anti-inflammatory cytokine that modulates immune homeostasis [40,41,42]. Wang et al. [15] reported that dietary glucose oxidase supplementation downregulated the mRNA expression of pro-inflammatory cytokines, including duodenal TNF-α and ileal IL-6 in challenged piglets, thereby alleviating intestinal inflammatory injury. Chen et al. [10] demonstrated that dietary supplementation with the combination of GOD and macleaya extract decreased serum IL-1β concentration in piglets. In addition, we found that dietary GOD addition suppressed the relative gene expression of *NF-κB*, which was considered a key inducer of inflammation. *NF-κB* plays a pivotal role in orchestrating the inflammatory process. It responds to pro-inflammatory cytokines such as IL-1β, IL-6, and TNF-α, and controls the expression of multiple inflammatory mediators [43]. Therefore, dietary GOD supplementation mitigated systemic and placental inflammation, thereby improving the immune status and inhibiting the *NF-κB* expression in the placenta of farrowing sows.

An imbalance between pro-oxidant production and endogenous antioxidant defenses can disrupt redox homeostasis and impair normal physiological function in animals [44]. Severe disturbance of this balance may compromise immune function, stimulate inflammatory responses, and increase the risk of various disorders, thereby negatively affecting the health of sows [45]. It was reported that under intermediate oxidative stress, the *NF-κB* pathway could be activated [46]. Maternal and placental antioxidant status are closely interrelated. The increasing metabolic demands during fetal development may disturb maternal redox balance and reduce systemic antioxidant stability [47]. A decrease in maternal antioxidant capacity may further disrupt placental antioxidant balance and adversely affect placental function [45]. GOD may improve systemic antioxidant capacity through the activation of the intestinal antioxidant defense system, mediated by the low levels of H_2_O_2_ produced during the catalytic reaction of GOD [48]. In this study, GOD supplementation increased serum SOD activity and placental GSH-Px and CAT activities, and elevated the relative gene expressions of *GPX1*, *SOD1*, *SOD2*, and *CAT*, accompanied by reduced serum and placental MDA concentrations. MDA is a major byproduct formed during lipid peroxidation of polyunsaturated fatty acids and serves as the most frequently measured biomarker of oxidative stress [49]. SOD serves as the first line of antioxidant defense by converting superoxide anions to H_2_O_2_, and GSH-Px and CAT further scavenge H_2_O_2_ and lipid peroxides to prevent oxidative damage [17]. Consistently, Sun et al. [17] reported that dietary 60 U/kg GOD reduced oxidative stress with decreased plasma MDA level at day 1 of lactation in sows and at weaning in piglets. You et al. [50] also found that dietary GOD supplementation elevated total antioxidant capacity and the activities of GSH-Px and SOD, while decreasing MDA level in sows and piglets. *Nrf2*, a key transcription factor, plays an essential role in regulating the activity of endogenous antioxidant enzymes to counteract oxidative stress [51]. Increasing the mRNA expression level of *Nrf2* can enhance the activities of antioxidative enzymes [52]. Additionally, *Nrf2* regulates the activity of *HO-1*, an important antioxidative enzyme that modulates intracellular ROS levels and serves as a sensitive and reliable indicator of cellular oxidative stress [53]. In the present study, we also found that dietary GOD supplementation increased relative mRNA expressions of *Nrf2* and *HO-1* in the placenta of sows. Previous study in weaned piglets indicated that GOD addition increased the antioxidant enzyme activities through upregulating hepatic and intestinal *Nrf2* signaling pathway [54]. Rost et al. [55] showed that nontoxic concentrations of H_2_O_2_ produced by GOD resulted in hepatic *HO-1* expression in rats. Therefore, the enhanced placental antioxidative capacity might be attributed to the upregulation of the *Nrf2*/*HO-1* signaling pathway induced by GOD supplementation. Above all, dietary inclusion of 300 mg/kg GOD improved antioxidant defense in sows, which may be helpful in enhancing immune function and health in sows and the placenta.

The gut microbiota plays a crucial role in regulating host physiological metabolism, immune function, and oxidative stress responses [48]. In the present study, dietary GOD supplementation had no significant impact on the α diversity of fecal microbiota in farrowing sows, which was consistent with the findings of Dang et al. [14] in weaning pigs fed an Aspergillus niger-expressed GOD-supplemented diet. However, GOD supplementation changed the β diversity of fecal microbiota of sows before farrowing in this study, demonstrating that GOD could reshape community structure. Previous study in piglets also reported that the combination of macleaya extract and GOD modified the cecal microbial β diversity [10]. Regarding the relative abundance at the phylum level, Bacillota (Firmicutes) and Bacteroidota (Bacteroidetes) were the most predominant phyla in the fecal microbiota of sows in both groups, which was consistent with previous studies in sows [56]. In addition, in the present study, GOD supplementation significantly reduced the relative abundance of *Clostridium*, *dga-11_gut_group*, *Bacteroides*, and *Prevotellaceae_NK3B31_group* while increasing the relative abundance of *Lachnospira*, *unclassified_f_Erysipelotrichiaceae*, and *Anaerostipes*, which could be attributed to several regulatory effects of GOD. Pathogenic strains of *Clostridium* have been reported to disrupt intestinal barrier integrity, trigger intestinal and hepatic oxidative stress, and compromise host health [57]. GOD exerts antimicrobial effects through the enzymatic generation of H_2_O_2_, which inhibits the proliferation of pathogenic bacteria [58]. Meanwhile, the gluconic acid, another catalytic product of GOD, reduces intestinal pH [58]. It has been reported that the abundance of Bacteroidetes decreased with the decrease in pH [59], while *Prevotellaceae_NK3B31_group*, *Bacteroides*, and *dga-11_gut_group* belong to Bacteroidetes. Michiels et al. [60] reported that butyric acid in the intestine increased with the gluconic acid supplementation level. *Lachnospira* and *Anaerostipes* are members of Lachnospiraceae, which could regulate inflammation by butyric acid mediation [61]. The two genera were reported to improve gut barrier integrity, enhance immune surveillance, alleviate inflammation and oxidative stress, and confer a certain degree of resistance to environmental toxins [62]. Gluconic acid also promotes lactate production in the foregut, potentially reducing substrates for propionate production in the hindgut, thereby inhibiting the proliferation of propionate-producing bacteria such as *Prevotellaceae_NK3B31_group* and *Bacteroides* [61]. This phenomenon may reflect a shift in the cross-feeding ecology of gut microbiota towards secondary metabolites induced by GOD. Studies also showed that *dgA-11_gut_group* was negatively correlated with plasma IgM concentrations, which could be a possible explanation for the increased serum IgM in the present study [23]. Previous studies have demonstrated that salivary cortisol is positively correlated with the relative abundance of *Prevotellaceae_NK3B31_group* and *Bacteroides*, with which sows showed better reproductive performance and health status [20,63]. Additionally, the abundance of *Bacteroides* was shown to be positively associated with endometritis and negatively with arginine metabolism, which was conducive to the protein genesis in the mammary gland [64,65]. Studies on *Erysipelotrichiaceae* are limited, but *Erysipelotrichiaceae CCMM* exhibited negative correlations with negative emotional indicators in sows, such as fatigue, depression, and anxiety states, suggesting it may participate in regulating stress response, potentially conferring benefits for gestation and farrowing [66]. Therefore, the alteration of fecal bacterial structure indicated beneficial effects of GOD supplementation on prepartum sows, which was beneficial to the resistance of pathogens during farrowing and the reason for reduced inflammation cytokines.

## 5. Conclusions

In this study, dietary supplementation with 300 mg/kg glucose oxidase during mid-to-late gestation enhanced antioxidant capacity and immune function, suppressed systemic and placental inflammation. These improvements were accompanied by a modulated gut microbiota, with an increased abundance of beneficial bacteria and a reduced abundance of potentially pathogenic bacteria. However, these findings were obtained under controlled experimental conditions; further validation in large-scale commercial production systems is warranted. Additionally, while this study showed the beneficial effects of glucose oxidase at farrowing, the long-term implications for reproductive performance and offspring health remain to be explored. Overall, supplementation with glucose oxidase improved the health status of sows at farrowing.

## Figures and Tables

**Table 1:** Ingredients and nutrient levels of basal diets of sows during gestation (as-fed basis).

Items	Content
Ingredients, %	
Corn	44.00
Barley (naked)	27.50
Soybean meal (46%)	9.00
Rice bran meal	6.00
Citric acid residue	5.50
Alfalfa hay	3.50
Premix ^1^	4.00
Lysine (70%)	0.50
Nutritional Composition ^2^, %	
DE, kcal/kg	3140.30
Crude protein	14.42
Crude fat	2.69
Crude fiber	4.60
Calcium	0.90
Available P	0.35
Lysine	0.92
Methionine	0.22
Threonine	0.61
Tryptophan	0.16
Valine	0.68

^1^ Provided per kg of diet: vitamin A 6608 IU, vitamin D3 1652 IU, vitamin E 27.5 IU, vitamin K 4.4 mg, Fe 100 mg, Cu 6.6 mg, Mn 30 mg, Se 0.15 mg, Zn 100 mg, I 0.6 mg, thiamine 1.66 mg, riboflavin 6.6 mg, niacin 40 mg, pantothenic acid 25 mg, vitamin B12 33 μg, pyridoxine 0.8 mg, folic acid 1.5 mg, biotin 0.22 mg, choline 583 mg. ^2^ Nutrient levels were the total calculated nutrient values.

**Table 2:** Primers used in real-time quantitative PCR.

Gene	Primer	Sequence (5′ → 3′)	GenBank ID
*Nrf2*	Forward	CCAGTCTTCATTGCTCCTAACCA	XM_021075133.1
	Reverse	CCTCCCAAACTTGCTCAATATCCT	
*GPX1*	Forward	TCTCCAGTGTGTCGCAATGA	NM_214201.1
	Reverse	TCGATGGTCAGAAAGCGACG	
*CAT*	Forward	CCTGCAACGTTCTGTAAGGC	NM_214301.2
	Reverse	GCTTCATCTGGTCACTGGCT	
*SOD1*	Forward	AGACCTGGGCAATGTGACTG	NM_001190422.1
	Reverse	GTGCGGCCAATGATGGAATG	
*SOD2*	Forward	AATCTGAGCCCTAACGGTGG	NM_214127.2
	Reverse	GGCTTCCAGCAATTCCCCTTT	
*NF-κB*	Forward	TCGCTGCCAAAGAAGGACAT	NM_001048232.1
	Reverse	TAGCGTTCAGACCTTCACCG	
*HO-1*	Forward	TGATGGCGTCCTTGTACCAC	NM_001004027.1
	Reverse	GACCGGGTTCTCCTTGTTGT	
*β-actin*	Forward	TCAGCAAGCAGGAGTACGAC	NM_001444420.1
	Reverse	AATGCAACTAACAGTCCGCC	

*Nrf2*, Nuclear factor erythroid-derived 2-like 2; *GPX1*, Glutathione peroxidase 1; *CAT*, Catalase; *SOD1*, Superoxide dismutase 1; *SOD2*, Superoxide dismutase 2; *NF-κB*, Nuclear factor kappa-B; *HO-1*, Heme oxygenase 1.

**Table 3:** Effects of GOD supplementation during gestation on serum biochemistry of farrowing sows.

Items	CON	GOD	SEM	*p*-Value
ALT (U/L)	37.17	29.60	1.76	0.023
AST (U/L)	38.67	41.20	2.73	0.668
ALP (U/L)	39.60	49.00	3.02	0.125
LDH (U/L)	451.67	478.40	25.38	0.626
TP (g/L)	67.52	68.73	1.89	0.764
ALB (g/L)	48.50	50.15	1.02	0.443
GLU (mmol/L)	5.57	5.83	0.27	0.642
BUN (mmol/L)	3.53	4.59	0.29	0.062
CHOL (mmol/L)	1.46	1.93	0.15	0.115
TG (mmol/L)	0.26	0.60	0.07	0.002

CON, sows fed a basal diet; GOD, sows fed a basal diet supplemented with 300 mg/kg GOD. ALT, alanine aminotransferase; AST, aspartate aminotransferase; ALP, alkaline phosphatase; LDH, lactate dehydrogenase; TP, total protein; ALB, albumin; GLU, glucose; BUN, blood urea nitrogen; CHOL, cholesterol; TG, triglyceride. *n* = 6. SEM, standard error of the means. *p* < 0.05 was considered as significant difference.

**Table 4:** Effects of GOD supplementation during gestation on serum immunoglobulins of farrowing sows.

Items (μg/mL)	CON	GOD	SEM	*p*-Value
IgM	566.70	702.76	27.73	0.005
IgA	13.39	17.74	1.46	0.149
IgG	1574.32	1558.26	107.53	0.945

CON, sows fed a basal diet; GOD, fed a basal diet supplemented with 300 mg/kg GOD. IgM, immunoglobulin M; IgA, immunoglobulin A; IgG, immunoglobulin G. *n* = 6. SEM, standard error of the means. *p* < 0.05 was considered a significant difference.

**Table 5:** Effects of GOD supplementation during gestation on serum and placental inflammatory cytokine levels of farrowing sows.

Items	CON	GOD	SEM	*p*-Value
Serum (pg/mL)				
IL-1β	58.34	46.94	4.70	0.242
IL-6	46.02	38.38	1.89	0.032
IL-10	40.51	62.95	5.34	0.020
TNF-α	261.16	205.98	23.95	0.269
Placenta (ng/g prot)				
IL-1β	31.58	18.64	2.79	0.009
IL-6	752.91	699.69	70.40	0.724
IL-10	142.94	151.01	8.35	0.651
TNF-α	445.60	260.65	34.00	0.001

CON, sows fed a basal diet; GOD, sows fed a basal diet supplemented with 300 mg/kg GOD. IL-1β, interleukin-1β; IL-6, interleukin-6; IL-10, interleukin-10; TNF-α, tumor necrosis factor-α. *n* = 6. SEM, standard error of the means. *p* < 0.05 was considered as significant difference.

**Table 6:** Effects of GOD supplementation during gestation on serum and placental antioxidant capacities of farrowing sows.

Items	CON	GOD	SEM	*p*-Value
Serum				
GSH-Px (U/mL)	2604.38	3178.13	167.28	0.084
CAT (U/mL)	2.72	2.97	0.55	0.830
SOD (U/mL)	591.92	679.99	22.51	0.041
MDA (nmol/mL)	3.29	1.46	0.50	0.059
Placenta				
GSH-Px (U/mg prot)	2.21	4.95	0.62	0.016
CAT (U/mg prot)	3.52	5.00	0.43	0.090
SOD (U/mg prot)	46.15	53.75	4.12	0.381
MDA (nmol/mg prot)	3.90	2.98	0.20	0.009

CON, sows fed a basal diet; GOD, sows fed a basal diet supplemented with 300 mg/kg GOD. GSH-Px, Glutathione Peroxidase; CAT, Catalase; SOD, Superoxide Dismutase; MDA, Malondialdehyde; *n* = 6. SEM, standard error of the means. *p* < 0.05 was considered as significant difference.

## Data Availability

The data presented in this study are available from the corresponding author upon reasonable request. The raw sequencing data (FASTQ files) have been deposited in the NCBI Sequence Read Archive under accession number PRJNA1442410 (Illumina sequences): https://www.ncbi.nlm.nih.gov/bioproject/PRJNA1442410 (accessed on 25 March 2026).
